# TCreERT2, a Transgenic Mouse Line for Temporal Control of Cre-Mediated Recombination in Lineages Emerging from the Primitive Streak or Tail Bud

**DOI:** 10.1371/journal.pone.0062479

**Published:** 2013-04-30

**Authors:** Matthew J. Anderson, L. A. Naiche, Catherine P. Wilson, Cindy Elder, Deborah A. Swing, Mark Lewandoski

**Affiliations:** 1 Cancer and Developmental Biology Lab, National Cancer Institute, Frederick, Maryland, United States of America; 2 Mouse Cancer Genetics Program, National Cancer Institute, Frederick, Maryland, United States of America; Pennington Biomedical Research Center/LSU, United States of America

## Abstract

The study of axis extension and somitogenesis has been greatly advanced through the use of genetic tools such as the TCre mouse line. In this line, Cre is controlled by a fragment of the *T (Brachyury)* promoter that is active in progenitor cells that reside within the primitive streak and tail bud and which give rise to lineages emerging from these tissues as the embryonic axis extends. However, because TCre-mediated recombination occurs early in development, gene inactivation can result in an axis truncation that precludes the study of gene function in later or more posterior tissues. To address this limitation, we have generated an inducible TCre transgenic mouse line, called TCreERT2, that provides temporal control, through tamoxifen administration, in all cells emerging from the primitive streak or tail bud throughout development. TCreERT2 activity is mostly silent in the absence of tamoxifen and, in its presence, results in near complete recombination of emerging mesoderm from E7.5 through E13.5. We demonstrate the utility of the TCreERT2 line for determining rate of posterior axis extension and somite formation, thus providing the first *in vivo* tool for such measurements. To test the usefulness of TCreERT2 for genetic manipulation, we demonstrate that an early deletion of ß-Catenin via TCreERT2 induction phenocopies the TCre-mediated deletion of ß-Catenin defect, whereas a later induction bypasses this early phenotype and produces a similar defect in more caudal tissues. TCreERT2 provides a useful and novel tool for the control of gene expression of emerging embryonic lineages throughout development.

## Introduction

The Cre/loxP system of site-specific DNA recombination, modified from bacteriophage P1 to function in the murine milieu, is now a standard approach to control gene expression in genetically modified mice [1], [2], [3]. For example, all strategies currently employed by the international mouse knockout project to inactivate every gene in the mouse genome uses Cre-mediated recombination in some aspect of the targeting strategy [4].

One extremely useful transgenic Cre mouse line is the TCre line [5]. In this line, Cre expression is regulated by a *T* (*Brachyury*) regulatory element, which is active in a subset of the endogenous *T* expression domain, in cells within and emerging from the primitive streak (PS) during gastrulation [6] and then, as the embryo extends posteriorly, in the tailbud during secondary axis extension [7], [8]. Because the primitive streak generates mesoderm and endoderm during gastrulation and the tailbud generates all three germ layers: mesoderm, neuroectoderm and endoderm [9], these lineages are recombined by TCre action [5], [10]. However, amongst the many studies where TCre has been used to control gene expression [10], [11], [12], [13], [14], [15], [16], [17], [18], [19], [20], [21], [22], [23], [24], [25], [26], there is a subset wherein the TCre-mediated defect strongly affects axis extension, resulting in an embryonic truncation that precludes investigation of more posterior regions of the embryo [12], [13], [16], [20], [23], [25]. For example, when TCre is used to inactivate or activate ß-Catenin, an essential component of “canonical” WNT signaling, the resulting embryos do not develop normally past embryonic day 8.5 (E8.5) [13], [16], [25].

To access and control gene expression in posterior regions of such mutant embryos, an inducible version of TCre is needed, so that temporal control can be added to the tissue-specific regulation provided by the Brachyury regulatory element driving Cre expression. One way to achieve this is to use a modified open reading frame that encodes a Cre protein fused with a modified ligand-binding domain of the estrogen receptor [27], [28]. Because translocation of this fusion protein to the nucleus is dependent on tamoxifen (Tam), Cre-mediated recombination is prevented until Tam is delivered to the Cre-expressing cells. Here, we describe the generation and characterization of one such mouse line, called TCreERT2, and characterize the recombination pattern generated by this line. We then demonstrate its utility through the Tam-dependent inactivation of ß-Catenin.

## Results and Discussion

### Generation and Characterization of the TCreERT2 Mouse Line

To create a Tam-inducible version of the TCre mouse line, we first generated a construct that was identical to that used to generate TCre [5] except that the open reading frame encoded CreERT2, the more sensitive version of the fusion construct in which the Cre coding region is fused with that of a mutated ligand-binding domain of the human estrogen receptor [29]. This construct was injected into B6C3F2 zygotes to generate a series of transgenic lines that were positive by PCR genotyping. After a preliminary screen of Cre activity in 8 transgenic mouse lines, we settled on one, called TCreERT2, which exhibited extensive Cre-mediated recombination upon Tam-induction but little to none in its absence (see Materials and Methods for details). Mice can be made homozygous for TCreERT2 with no obvious effects on health or fertility.

**Figure 1:**
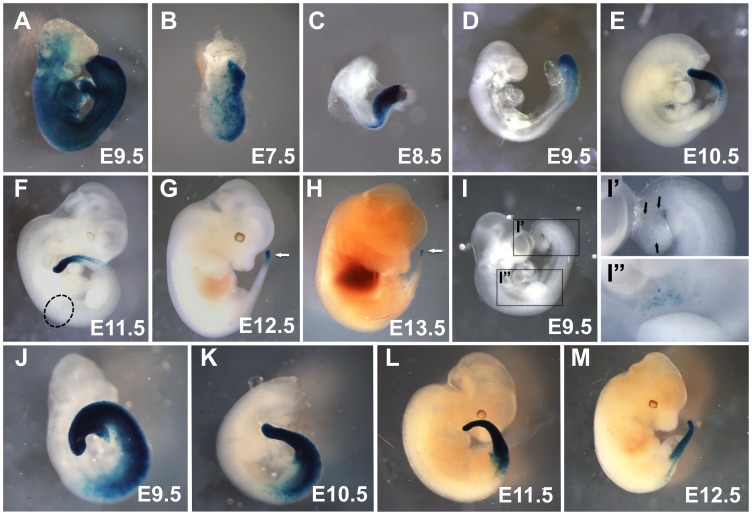
Tamoxifen adminstration induces TCreERT2-mediated recombination specifically in the primitive streak or tail bud. Embryos of the indicated age carrying either the TCre (**A**) or TCreERT2 transgenes (**B–M**) were assayed for activation of the *R26R* Cre-reporter by staining for ßGal activity (blue color). TCre; *R26R* control embryos (**A**) display widespread recombination at E9.5 compared to TCreERT2;*R26R* embryos, which, 24 hours after induction, have activated R26R only in tissues recently emerged from the primitive streak at E7.5 and E8.5 (**B, C**) or tailbud at E 9.5–13.5 (**D–H**) (white arrows in **G** and **H**). An uninduced E9.5 TCreERT2;*R26R* embryo displays almost no blue cells (**I**) except for two small clusters (**I’** and **I”,** which are enlargements of the boxed regions in **I;** arrows point to individual blue cells. A similar region is circled in **F**). 48 hours after induction, TCreERT2;*R26R* embryos from E9.5–E12.5 display a recombined region that extends more rostrally (**J–M**) than in the case of a 24-hour induction period.

To assess recombination activity of TCreERT2 throughout development, the Gt(ROSA)26Sortm1Sor (R26R) reporter line was utilized [30], allowing visualization of recombination by the Cre-mediated activation of a near-ubiquitously expressed lacZ gene, which encodes beta-galactosidase (ßgal). Female R26R homozygotes were mated to TCreERT2 males, given an intraperitoneal injection of Tam at specific times during pregnancy and then sacrificed 24 hours later for embryo harvest (Figure 1B–H). Such a 24-hour induction at E6.5 produced embryos with ßgal activity in the primitive streak (PS) and emerging mesoderm at E7.5 (Figure 1B). Induction with Tam at E7.5 yielded embryos with ßgal activity in the PS, presomitic mesoderm (PSM), and posterior somites at E8.5 (Figure 1C). Likewise, induction at later stages produced recombination of these tissues (Figure 1D–H) up until the end of somitogenesis at E13.5, the point at which axis extension ceases (Figure 1H). At all time points, recombination occurred only in the tissues recently emerging from the primitive streak at the most caudal end of these embryos, in sharp contrast to the widespread recombination caused by TCre (Figure 1A and [5], [10]). We then stained embryos at various embryological stages at 48 hours after induction. As expected, recombination was again restricted to the caudal end of the embryo, but now the border of the recombination domain was more anterior than in the 24-hour inductions (compare Figure 1J–M with 1D–G).

To determine the extent of “leaky” Cre expression in the absence of Tam induction, we stained five uninduced TCreERT2; R26R embryos, harvested at E9.5. Amongst these controls, we detected no blue cells in one embryo and the other four each had two very small domains of clustered blue cells, suggestive of a small clone of cells, in the tailbud region and in the flank mesoderm near the forelimb. One of the more extensively recombined control embryos is shown in Figure 1I–I”. Consistent with this miniscule level of Tam-independent Cre activity, we occasionally saw a few blue cells in the flank mesoderm of experimental (Tam-induced) embryos (circle in Figure 1F).

We also tested whether Cre could be induced in adult tissues by administering a single dose of Tam to 1.5–3 month old TCreERT2; R26R males and females, and then after 24 or 60 hours harvesting and staining the following organs: brain, heart, lung, liver, spleen, stomach, kidneys, intestines, testes, uterus and ovaries. No blue cells were detected in these organs (data not shown). Our embryonic and adult data considered together, show that TCreERT2 activity is dependent on Tam induction and is restricted to the newly formed caudal tissues of the embryo during embryogenesis.

**Figure 2:**
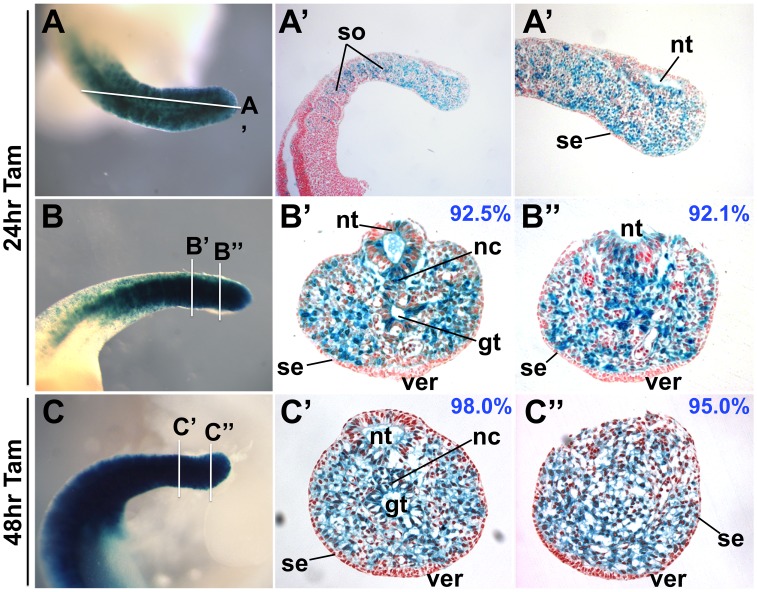
TCreERT2 activation results in high levels of recombination. Sagittal (**A–A’**) or transverse (**B–B”, C–C”**) sections of an XGal- stained TCreERT2;*R26R* E10.5 embryo 24 (**A–B”**) or 48 hours (**C–C”**) after tamoxifen induction, illustrates the extent of recombination (blue cells). Lines in A, B and C illustrate the approximate section locations. Blue numbers indicate the percentage of blue mesodermal cells (excluding blood cells), which increase slightly after a 48-hour induction period from about 92% to 95–98% (**C’** and **C”**). Note the near absence of blue cells in the surface ectoderm (se). gt, gut tube; nc, notochord; nt, neural tube; so, somites; ver, ventral ectodermal ridge.

**Figure 3:**
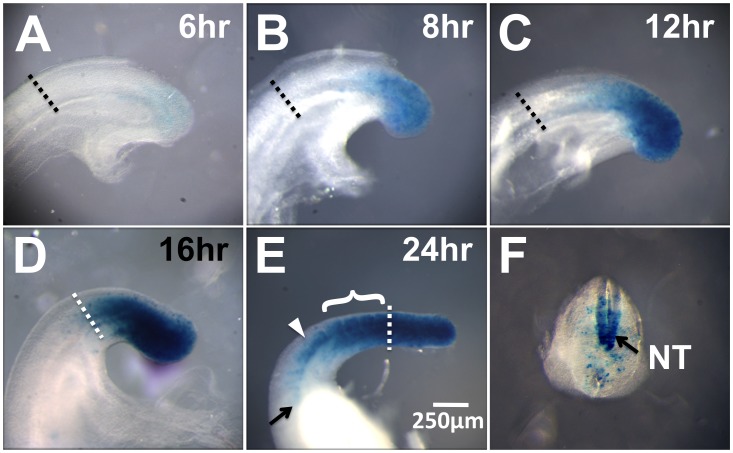
TCreERT2 recombination dynamics allows measurement of the *in* *vivo* rate of axis extension and somitogenesis. A single dose of tamoxifen was injected at 8∶00 AM to dams carrying an E9.0–E9.5 TCreERT2; R26R litter. After the indicated time interval, embryos were harvested and stained for ßGal activity (**A–E**). The dotted line in all panels marks the border between the anterior PSM and the most caudal somite. After 6 hours, blue cells can just be discerned (**A**). The intensity of blue increases progressively by 8 hours (**B**) and 12 hours (**C**), indicating an increase in the fraction of recombined cells. By 16 hours, the recombined domain has reached the border between the anterior PSM and youngest somite (**D**). Over the next 8 hours, the four most caudal somites (bracket in **E**), which together measure approximately 470 µm, are heavily labeled (24-hour point**, E**) demonstrating that, at this stage, the embryo is extending about one µm/min (470 µm/480 min) and a somite forms about every 2 hours. In the lateral view in **E** it appears that somites rostral to the bracket are labeled (i.e, there are more than 4 blue somites), but this is an illusion due to viewing a blue labeled neural tube (NT) through translucent, mostly unlabeled, somites. This is demonstrated by a transverse view, shown in panel **F**, of the embryo in **E** (white arrowhead **E** indicates where embryo was sliced).

To determine the extent of TCreERT2-mediated recombination, we sectioned E10.5 tailbuds that had been stained for ßgal activity at 24 or 48 hours post Tam induction. In addition to extensive recombination in the early mesoderm, recombination was also observed in the neural and gut tubes, but virtually absent from the surface ectoderm, including the ventral ectodermal ridge (VER, Figure 2). The labeling of these diverse tissues indicates that TCreERT2 is expressed in a tailbud stem cell population required for the coordinated extension of the body axis. This domain presumably includes, but is larger than, the chordoneural hinge [9], a common source of progenitors for the mesodermal and neuroectodermal lineages. Within the neural tube, TCreERT2 acts more readily on lineages fated to become ventral as compared to dorsal neural tube (Figures 2B’, B”, C’, 3F). At 24 hours post-induction these labeled ventral neural tube cells can be observed to extend anteriorly beyond the recombined somitic mesoderm (Fig. 3E,F). To estimate the amount of recombination in the mesoderm, we counted blue vs. non-blue cells in these sections and found that about 92% of mesodermal cells were recombined 24 hours after induction (Figure 2B–B”; representative of 4 sectioned embryos); this value increased to 95–98% by 48 hours (Figure 2C–C” representative of 3 sectioned embryos). These high levels of recombination are reproducibly achieved when a maximum Tam dosage is delivered; however, we note that lower doses can result in a small subset of recombination, which can be useful in lineage tracing experiments (data not shown).

### Determining the Rate of *in vivo* Axis Extension and Somitogenesis

The TcreERT2 transgene expresses Cre recombinase in the progenitors of the presomitic mesoderm (Figure 1), and the descendents of these cells become incorporated into the embryo as the axis extends. Therefore, by carefully analyzing the onset of R26R activation after a single Tam pulse, we could follow these labeled lineages over a defined time period and thereby obtain an in vivo estimate of the rate of both axis extension and somitogenesis. This is illustrated in Figure 3, which shows E10.5 tailbuds stained for activated R26R ßgal activity, at 6, 8, 12, 16 and 24 hours after Tam induction. ßgal activity starts appearing in the posterior PSM 6 hours after Tam induction (Figure 3A), consistent with previous reports using other CreERT drivers [31]. During the next 6 hours this domain expands as the axis extends (Figure 3B, C) and by 16 hours after Tam administration, the ßgal+domain abuts the border between the PSM and the most posterior somite (Figure 3D, dotted line).

Over the next 8 hours, (that is, 24 hours after TAM induction) we found that approximately four somites were labeled (3.94±0.217 (SEM), n = 17). This provides a rate of somite formation of 2 hours per somite, confirming previous observations [32], [33]. In a representative image (Figure 3E), we note that these four labeled somites measure approximately 470 µm. Thus, 470 µm of the mesoderm beyond the border between the PSM and the most posterior somite is labeled in an 8 hour period, providing an extension rate of about a µm each minute. The current method for such measurements involves in vitro incubation of the tail bud for a defined time period [34], which may be subject to artifacts due to culture conditions. Such artifacts are avoided if TCreERT2 is used as an *in vivo* measuring tool for determining the rate of axis extension and somite formation between and within litters.

### Using TCreERT2 to Bypass Early Lethality Due to Gene loss-of-function

To demonstrate the utility of TCreERT2 activity for bypassing early lethality and studying gene function in later development, we deleted the gene encoding *ß-Catenin (Ctnnb1)* at two different time points with TCreERT2. It has been previously shown that deletion of *ß-Catenin* in the conventional TCre mouse results in a severely truncated embryo that does not develop much beyond E8.5 (when the six most anterior somites would form in normal control embryos) and the mutant somites that do form are small and disorganized [13], [16]. This phenotype is due, in part, to the loss of the Wnt3a/ß-Catenin target gene *Msgn1* [25].

**Figure 4:**
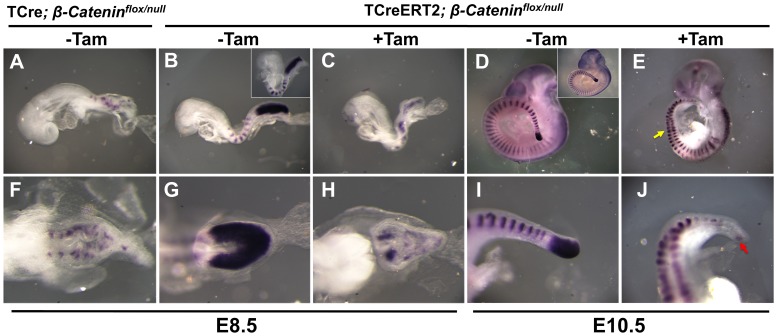
TCreERT2-mediated deletion of *ß-catenin* at different axial levels. All embryos shown, of the indicated genotype and stage, with or without a 48-hour Tam induction, were stained for expression of the markers *Uncx4.1* and *Msgn1*. Both markers were developed with the same color reaction, but they mark mutually exclusive domains: *Uncx4.1* is expressed only in stripes in the somites and *Msgn1* is expressed only in the PSM. **A–E** are lateral views and **F–J** are either dorsal views (**F–H**) or magnified views of the caudal end of the embryo immediately above (**I, J**). TCre-mediated deletion of *ß-catenin* causes caudal truncation and disorganized somites at E8.5 (**A, F**). These E8.5 defects are phenocopied in the Tam-induced TCreERT2; *ß-catenin ^flox/null^* embryos (**C,H**) but not in the uninduced control (**B,G**), which is similar to a normal TCreERT2; *ß-catenin ^flox/wt^* embryo (insert in **B**). Tam induction can produce similar somitogenesis defects later in E10.5 TCreERT2; *ß-catenin ^flox/null^* embryo (**E, J**). The yellow arrow in **E** indicates the axial level where caudal somite defects begin as indicated by *Uncx4.1* staining. The red arrow in **J** points to residual *Msgn1* expression. Uninduced E10.5 TCreERT2; *ß-catenin ^flox/null^* control embryos (**D, I**) are similar to TCreERT2; *ß-catenin ^flox/wt^* embryos (insert in **D**).

We first examined E8.5 mutants in which *ß-Catenin* was inactivated by a Tam induction of TCreERT2 at E6.5 (Figure S1). The resulting defect (Figure 4C,H) largely phenocopies the defect caused by TCre-mediated inactivation (Figure 4 A, F and [13], [16]). Staining for *Uncx4.1*, which marks the posterior half of each somite [35], [36], reveals that somotigenesis is similarly disrupted in both TCre and TCreERT2-mediated mutants. We then examined TCreERT2; *ß-Catenin ^flox/null^* mutants at E10.5 induced with Tam at E8.5 (Figure 4E and J), a stage unavailable in TCre *ß-Catenin ^flox/null^* mutants due to earlier axis truncation [13], [16]. These mutants resembled those harvested at E8.5 in that *Msgn1* and *Uncx4.1* staining revealed, respectively, a depletion of the PSM and a disruption of somite patterning. Notably, disruption of somitogenesis occurred only in the most posterior somites, starting at about somite 14/15 (yellow arrow Figure 4E). This is as expected because the cells that gave rise to these somites resided in the PSM at the initiation of Cre action via TAM induction.

### Concluding Remarks

The TCreERT2 mouse line is useful for the Cre-mediated control of gene expression in lineages emerging from the primitive streak and the tail bud at any stage of axis extension. A limitation specific to this line is indicated by the incomplete recombination of the R26R reporter in the dorsal neural tube. However, we caution any investigator to monitor recombination of their gene of interest because it is clear that not all genomic Cre targets are recombined with the same efficiency [37], [38].

There are currently several trangenic mouse lines in which Cre is expressed in a subset of these tissues but, in contrast to TCreERT2, have no temporal control over activity: TCre is active in most early PS progenitors (recombination is incomplete in the heart) [5], Dll1-Cre is active in a subset of these tissues, the anterior PSM, lateral plate and intermediate medoserm [39], and Meox1Cre activity is restricted to the paraxial mesoderm [40]. The only other Cre line that provides temporal control in this region of the embryo is Tbx6;CreER^Tg5769^, which is expressed in the anterior PSM but not the PS or posterior PSM [41]. Tbx6;CreER^Tg5769^-mediated recombination is therefore useful for studying genes required in somite maturation but is inadequate for studying genes required for processes occurring more posteriorly, such as somitogenesis and PSM maintenance. Conversely, the T promoter driving CreERT2 is expressed in the PS and PSM making genetic control in these progenitors accessible. The ability for temporal control provided by TCreERT2 allows one to abrogate gene expression in early mesoderm progenitors or their derivatives throughout development. This provides the opportunity to determine gene function within the PSM and its derivatives at all developmental stages.

## Materials and Methods

### Ethics Statement

This study was carried out in strict accordance with the recommendations in the Guide for the Care and Use of Laboratory Animals of the National Institutes of Health. The protocol was approved by the Animal Care and Usage Committee of NCI-Frederick (NIH) (Animal Study Proposal: 11-069).

### Generation of TCreERT2

To construct TCreERT2 we first restricted pML78 (which contains the ßactin-Cre construct described in Lewandoski et al [42]) with Xba1 and EcoR1, which yields a pBluescript backbone that contains an approximately 800 bp fragment that encodes the human ßactin polyA region. Into these sites we inserted an Xba-EcoR1 fragment that contains the 600 bp T promoter. We then opened this new plasmid at its unique EcoR1 site and inserted an EcoR1 fragment that contains the CreERT2 coding region, isolated from a plasmid kindly provided by Daniel Metzger. A plasmid with the CreERT2 in the correct orientation was the final construct. A Kpn-Xba1 digestion of this plasmid yielded a fragment containing the TCreERT2-human ßactin polyA construct, which was injected into B6C3F2 zygotes derived from intercrossing B6C3F1(C57BL/6NCr X C3H/HeNCrMTV-) mice.

These injections resulted in 227 mice, which were genotyped using primers that identified TCreERT2. These are located in the T promoter (T1) and within Cre (Cre2), generating an ∼800 bp product; T1: 5′ GGG ACC CAT TTT TCT CTT CC, and Cre2: 5′ CCA TGA GTG AAC GAA CCT GG. This analysis yielded 39 potential founder mice. Of these, 14 males were bred and 12 of these transmitted TCreERT2 to their progeny, establishing stable lines. Of these, 8 were tested for Cre activity by mating TCreERT2 males to females homozygous for the Cre reporter, *R26R* [30]. Pregnancies were timed by monitoring plugs and potentially pregnant dams were induced with a single tamoxifen injection. This initial screen resulted in our selecting one line, which we named TCreERT2.

### Mouse Lines

Mice were kept on a mixed background consisting of CD1/129SvEv/C57BL/6. Genotyping was performed as previously reported for the following alleles or transgenes: R26R [30], Ctnnb1^tm2Kem^ (*ß-catenin ^flox^*) [43] and TCre [5]. To genotype TCreERT2 we used the same primers as TCre. We intend to deposit the TCreERT2 with Jackson Laboratories for distribution to the mouse research community.

### Tamoxifen Administration

A tamoxifen stock solution was made by diluting tamoxifen powder (SIGMA T-5648) in corn oil (SIGMA C-8267) to a final concentration of 20 mg/mL and stored at 4°C for up to 4 weeks. Before use, the tamoxifen stock was heated to 37°C. Tamoxifen was injected intraperatonealy at 0.175 mg/g mouse weight or 8.75 uL of the stock solution per gram of mouse.

### Marker Analysis

In situ hybridization and Xgal staining for ßgal activity were performed as previously described [20]. Unless otherwise noted, at least three embryos were analyzed for each marker/stage.

## Supporting Information

Figure S1 **Efficient** **TCreERT2-mediated deletion of** ***ß-catenin*** **in E8.5 embryos.** After a 48-hour Tam induction, embryos were hybridized with a probe for the *ß-catenin* sequences that are deleted by Cre. Compared to the control genotype (top panel), TCreERT2; *ß-catenin ^flox/null^* embryos display an efficient deletion of *ß-catenin* (bottom panel). When this experiment is performed with embryos harvested at E10.5, after a similar 48-hour Tam induction, the *ß-catenin* probe results in a signal that is too weak in control embryos to be meaningful in experimental TCreERT2; *ß-catenin ^flox/null^* embryos.
(TIF)
