# Supplementary figures and images for: TCreERT2, a Transgenic Mouse Line for Temporal Control of Cre-Mediated Recombination in Lineages Emerging from the Primitive Streak or Tail Bud
Source: PLoS One. 2013 Apr 30;8(4):e62479. doi: 10.1371/journal.pone.0062479 (PMC3640045; doi:10.1371/journal.pone.0062479)

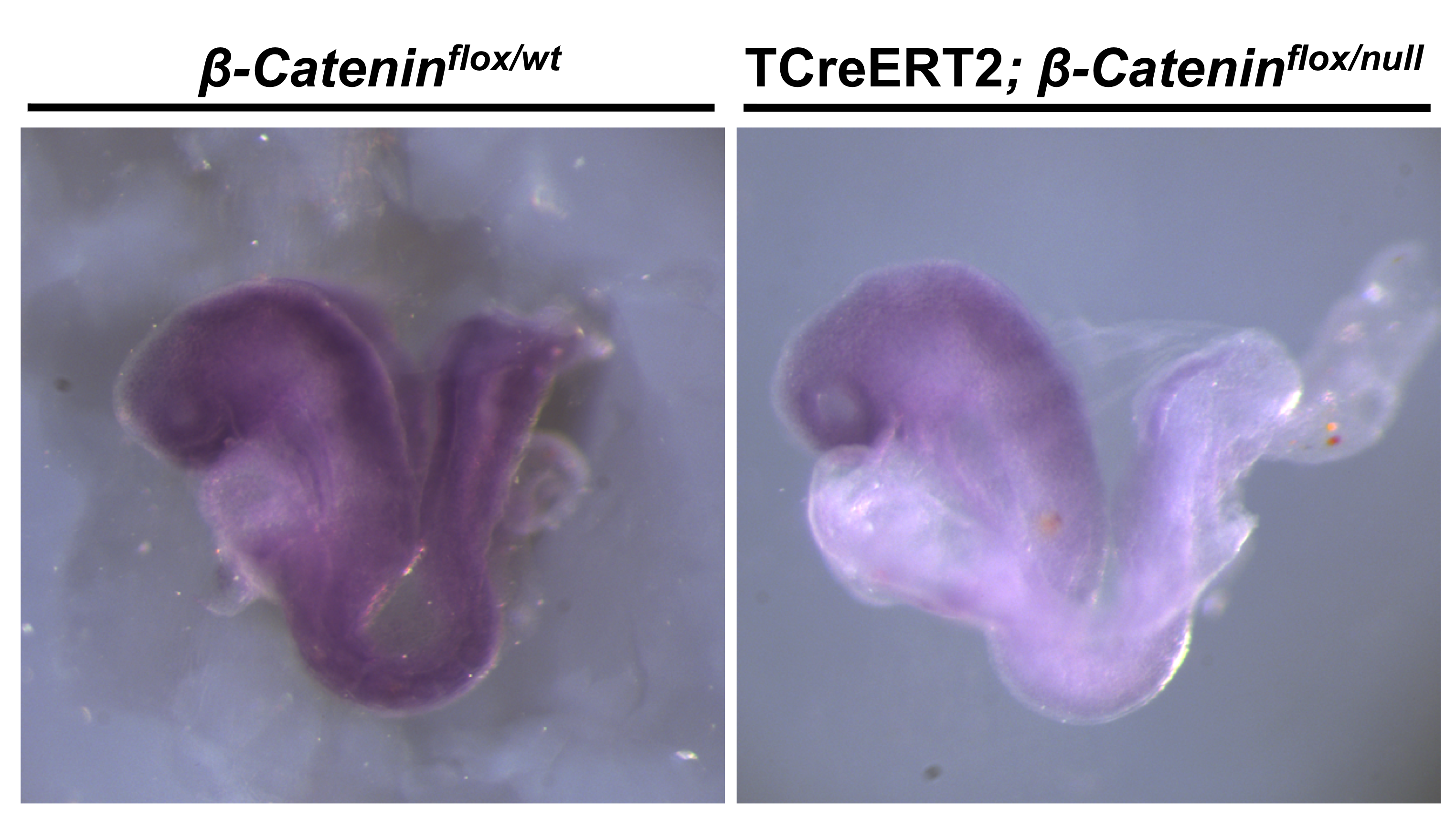

Supplement: Figure S1 — Efficient TCreERT2-mediated deletion of ß-catenin in E8.5 embryos. After a 48-hour Tam induction, embryos were hybridized with a probe for the ß-catenin sequences that are deleted by Cre. Compared to the control genotype (top panel), TCreERT2; ß-catenin flox/null embryos display an efficient deletion of ß-catenin (bottom panel). When this experiment is performed with embryos harvested at E10.5, after a similar 48-hour Tam induction, the ß-catenin probe results in a signal that is too weak in control embryos to be meaningful in experimental TCreERT2; ß-catenin flox/null embryos. (TIF) [file pone.0062479.s001.tif]
